# Whole-genome sequencing identifies a homozygous deletion encompassing exons 17 to 23 of the *integrin beta 4* gene in a Charolais calf with junctional epidermolysis bullosa

**DOI:** 10.1186/s12711-015-0110-z

**Published:** 2015-05-03

**Authors:** Pauline Michot, Oscar Fantini, Régis Braque, Aurélie Allais-Bonnet, Romain Saintilan, Cécile Grohs, Johanna Barbieri, Lucie Genestout, Coralie Danchin-Burge, Jean-Marie Gourreau, Didier Boichard, Didier Pin, Aurélien Capitan

**Affiliations:** INRA, UMR1313 Génétique Animale et Biologie Intégrative, domaine de Vilvert, Jouy-en-Josas, F-78352 France; ALLICE, 149 rue de Bercy, Paris, F-75012 France; Université de Lyon, VetAgro Sup, UPSP 2011-03-101 Interactions Cellules Environnement, 1 avenue Bourgelat, Marcy l’Etoile, F-69280 France; Cabinet des Vignes de la Fontaine, 41 rue du faubourg de Moulins, Saint-Pierre le Moutier, F-58240 France; UMR 1198 Biologie du Développement et Reproduction, domaine de Vilvert, Institut National de la Recherche Agronomique, Jouy-en-Josas, F-78352 France; INRA, UMR1388 GenPhySE, GeT-PlaGe, Castanet-Tolosan, F-31320 France; LABOGENA DNA, domaine de Vilvert, Jouy-en-Josas, F-78352 France; Institut de l’Elevage, 149 rue de Bercy, Paris 12, F-75595 France; Unité de Pathologie du Bétail, Ecole Nationale Vétérinaire d’Alfort, 7 avenue du Général de Gaulle, Maisons-Alfort, F-94704 France

## Abstract

**Background:**

Since 2010, four Charolais calves with a congenital mechanobullous skin disorder that were born in the same herd from consanguineous matings were reported to us. Clinical and histopathological examination revealed lesions that are compatible with junctional epidermolysis bullosa (JEB).

**Results:**

Fifty-four extended regions of homozygosity (>1 Mb) were identified after analysing the whole-genome sequencing (WGS) data from the only case available for DNA sampling at the beginning of the study. Filtering of variants located in these regions for (i) homozygous polymorphisms observed in the WGS data from eight healthy Charolais animals and (ii) homozygous or heterozygous polymorphisms found in the genomes of 234 animals from different breeds did not reveal any deleterious candidate SNPs (single nucleotide polymorphisms) or small indels. Subsequent screening for structural variants in candidate genes located in the same regions identified a homozygous deletion that includes exons 17 to 23 of the *integrin beta 4* (*ITGB4*), a gene that was previously associated with the same defect in humans. Genotyping of a second case and of six parents of affected calves (two sires and four dams) revealed a perfect association between this mutation and the assumed genotypes of the individuals. Mining of Illumina BovineSNP50 Beadchip genotyping data from 6870 Charolais cattle detected only 44 heterozygous animals for a 5.6-Mb haplotype around *ITGB4* that was shared with the carriers of the mutation. Interestingly, none of the 16 animals genotyped for the deletion carried the mutation, which suggests a rather recent origin for the mutation.

**Conclusions:**

In conclusion, we successfully identified the causative mutation for a very rare autosomal recessive mutation with only one case by exploiting the most recent DNA sequencing technologies.

**Electronic supplementary material:**

The online version of this article (doi:10.1186/s12711-015-0110-z) contains supplementary material, which is available to authorized users.

## Findings

Hereditary junctional epidermolysis bullosa (JEB) is a recessive inherited blistering disorder of the skin and mucous membrane in which tissue separation occurs within the lamina lucida (i.e. under the basal plasma membrane of the basal keratinocytes and above the basement membrane) of the basement membrane zone (BMZ) at the dermal-epidermal junction [1]. This rare mechanobullous disease was previously reported to be associated with mutations in genes encoding components of the hemidesmosome anchoring complex (*ITGA6*, *ITGB4*, *COL17A1*, and *LAMA3*, *LAMB3*, and *LAMC2*, encoding the subunit polypeptides of laminin 5) in humans and in several other mammalian species [2-8]. Congenital JEB has been sporadically observed over the past 30 years in Charolais cattle but, to date, the causative mutation has not been identified.

**Figure 1:**
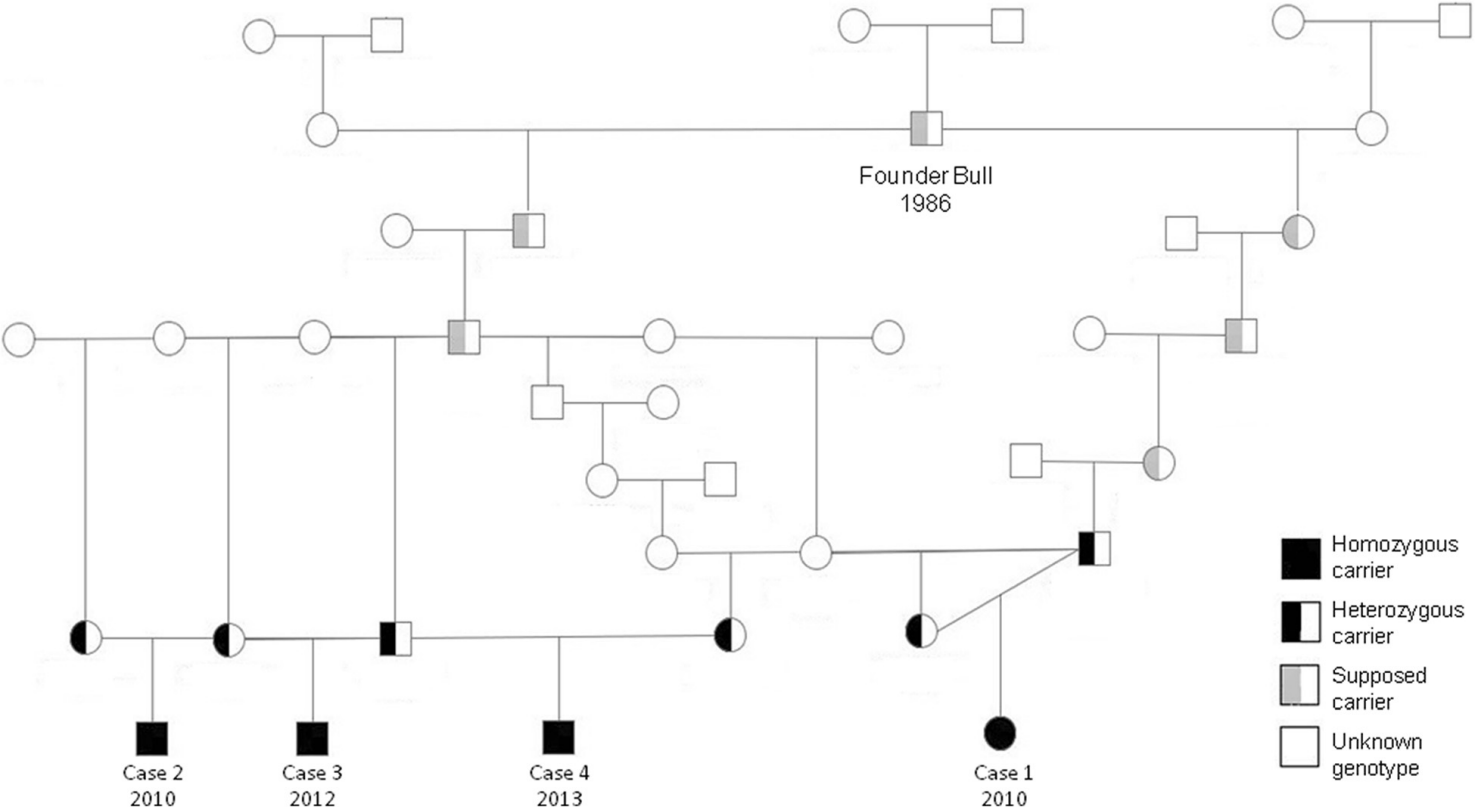
**Pedigree of Charolais cattle with JEB.** Note the high degree of consanguinity between the parents of male and female cases, which suggests an autosomal recessive mode of inheritance.

Since 2010, four cases (three males and one female) that were born in the same French herd from consanguineous matings were reported to us. Analysis of the pedigree data revealed that all JEB-affected animals trace back, on both the maternal and paternal sides, to a single founder bull born in Great Britain in 1986 (Figure 1), which suggests an autosomal recessive mode of inheritance as the most parsimonious model.

**Figure 2:**
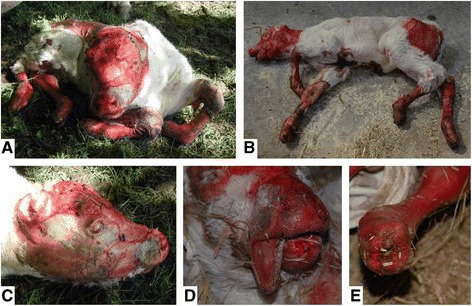
**Clinical features of recessive JEB in Charolais cattle. (A)** and **(B)** Global views of cases 3 and 2, respectively. **(C)** Head from case 3 showing atrophied pinna and skin lesions on the eyes, face and muzzle. **(D)** Lesions of the muzzle and tongue from case 1. **(E)** Forelimb from case 1 with dysungulation. These photos are personal photographs.

At birth, acral clinical lesions were observed with dysungulation of the four hooves and erosions and ulcers of the skin from the carpal and tarsal joints, fetlocks, ears, eyes, and muzzle and oral cavity (i.e. nares, tongue, buccal and labial sides of the mucosa and palate) (Figure 2). In addition, three of the four cases showed ear deformities (atrophied pinna and closed ears) and one displayed major epidermal loss on the back after being licked by its mother. General signs included anorexia, apathy, emaciation and marked cutaneous pain that justified rapid euthanasia.

**Figure 3:**
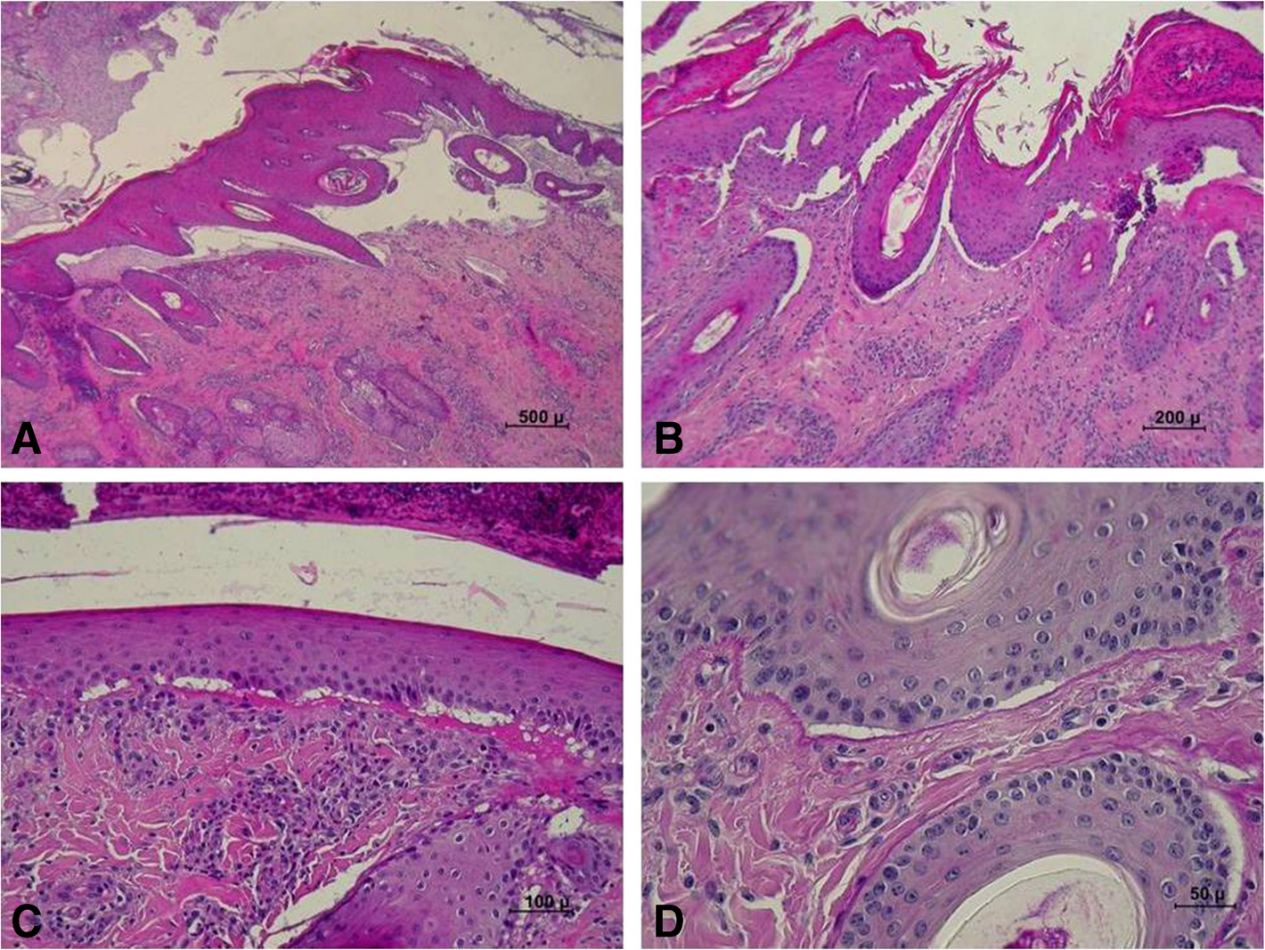
**Histopathological features of JEB in Charolais cattle. (A)** Large area of sub-epithelial splitting and blistering. **(B)** Sub-epithelial splitting and blistering, and cleft formation around hair follicles. **(C)** Vacuolation beneath basal keratinocytes of the epidermis and fibrin deposition on the dermal side of the vacuoles (5-μm section of tissue embedded in paraffin and stained with haematoxylin and eosin). **(D)** Vacuolation beneath basal keratinocytes of the epidermis and above the periodic acid Schiff (PAS)-positive basement membrane; 5-μm section of tissue embedded in paraffin and stained with PAS.

Histopathological examination of two cases revealed sub-epithelial splitting and blistering without keratinocyte cytolysis (Figure 3). The basal keratinocytes appeared to be intact. Periodic acid Schiff (PAS) staining was weakly positive for the basement membrane that was located at the base of the blisters. Cleft formation was sometimes present around hair follicles. Dermal inflammatory infiltrate was of varying degrees, very mild in non-ulcerated areas but marked in ulcerated areas.

Because of the rare occurrence of JEB in Charolais cattle and of rapid euthanasia of affected animals, only one case (out of the three that were born and reported at the beginning of the study) was available for DNA sampling, thus preventing the use of a classical autozygosity mapping approach [9,10]. As a consequence, we decided to sequence the whole genome (WGS) of this animal and applied an alternative strategy as described in Lupski et al. [11]. DNA was extracted from the pinna using the DNeasy Blood and Tissue Kit (Qiagen). One paired-end library with a 450-bp insert size was generated with the NEXTflex PCR-Free DNA Sequencing Kit (Bioscientific) and sequenced on one lane of the HiSeq 2000 platform (Illumina) with Illumina TruSeq V3 Kit (200 cycles).This has been submitted to the NCBI Sequence Read Archive under the accession number SRP055078. The 101-bp reads were mapped on the UMD3.1 bovine sequence assembly using BWA [12]. Reads with multiple alignments were removed (yielding a final average sequence coverage of 11.4 X) and variants were called using SAMtools [13].

**Figure 4:**
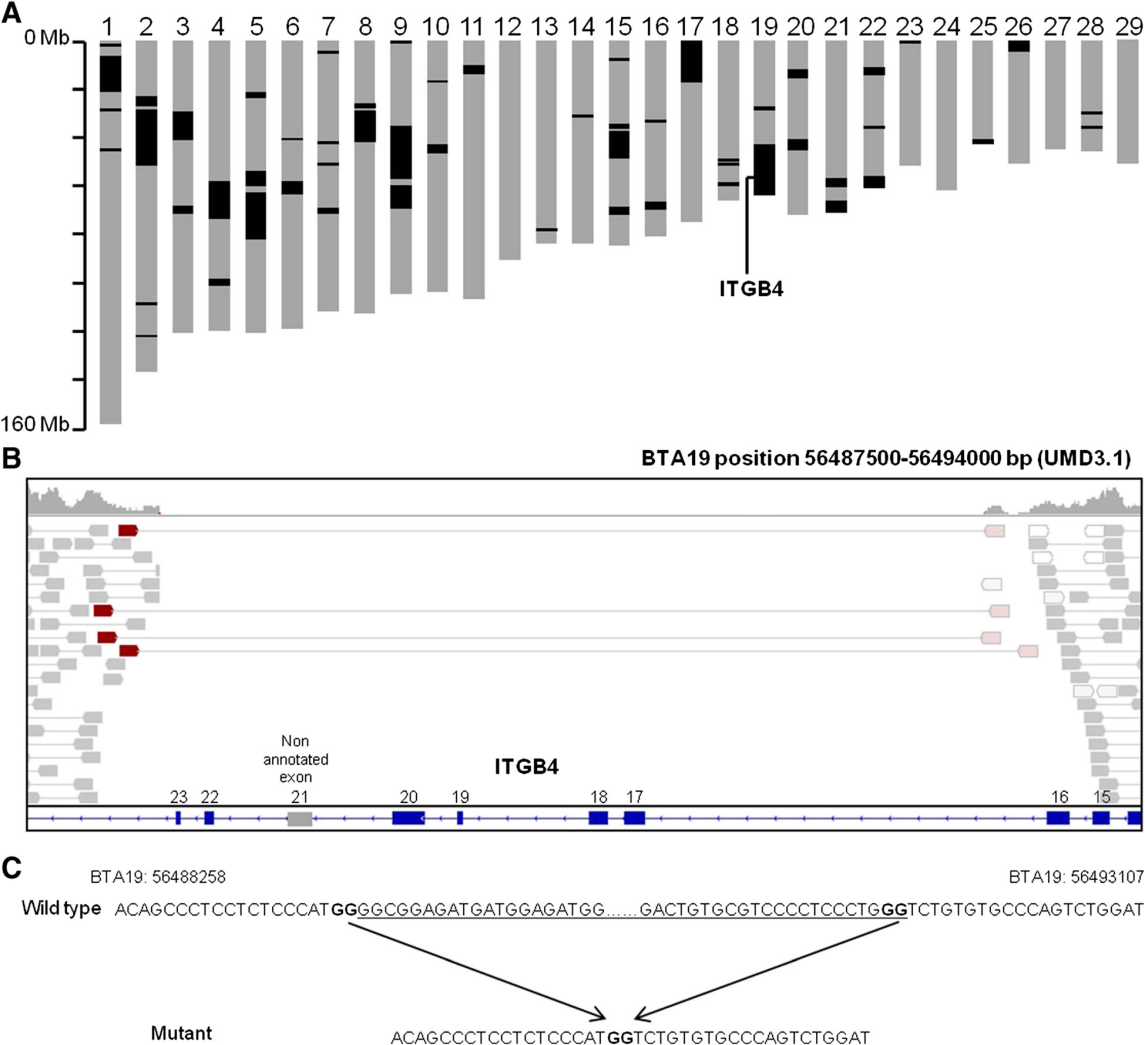
**Identification of the mutation responsible for JEB in Charolais cattle. (A)** Genome-wide screening for extended regions of homozygosity in one JEB-affected calf. Blocks of size ≥ 1 Mb are represented in black on each chromosome. **(B)** IGV snapshot showing the homozygous deletion that includes exons 17 to 23 of *ITGB4* in a JEB affected animal (modified based on results from the chromosome walking approach). **(C)** Details on the wild-type and mutant nucleotide sequences at the border of the deletion. The deleted segment is underlined; a GG doublet located on both sides of the deletion is indicated in bold.

Then, the genome of this consanguineous animal was screened for extended regions of homozygosity. To avoid artifactual heterozygous genotypes in homozygous regions due to pseudo-SNPs or sequencing errors, only 706 791 SNPs from the Illumina Bovine HD Beadchip were used. A total of 54 blocks with a minimal size of 1 Mb and containing only homozygous genotypes were identified (Figure 4A).

SNPs and small Indels located in these blocks and with a quality score greater than 30 were annotated using Ensembl VEP [14] and filtered for (i) homozygous polymorphisms observed in the WGS data from eight healthy Charolais animals and (ii) homozygous or heterozygous polymorphisms found in the genomes of 234 animals from different breeds [15] (assuming that the causative mutation is recessive and specific to the Charolais cattle). No homozygous deleterious mutation (frame-shifts, in-frame insertions or deletions, stop gain or loss of variants as well as polymorphisms that affect splice donor or splice acceptor sites and missense polymorphisms predicted to be deleterious) was found with this approach.

In a second attempt, we investigated the content of the homozygous blocks and found only one gene that was previously reported to be involved in JEB: *ITGB4*, which encodes the integrin beta 4 protein. A subsequent screening for structural variants using the Integrative Genomics Viewer (IGV) [16] enabled us to identify a 4.8-kb deletion on bovine chromosome 19 or BTA19 (g.56488278_56493087del on the UMD3.1 assembly). Since there were two gaps, and at least one artifactual segmental duplication in the current bovine assembly within the region deleted in JEB, we built a local assembly to determine the exact nature of the mutation, which finally consists in a 2831-bp deletion encompassing exons 17 to 23 of the *ITGB4* gene (Figure 4B, C and Additional file 1). This mutation was further confirmed by PCR-amplification of a 932-bp fragment that spans the deletion with the LEFT (TTCCCTGGGGGATCTGGGA) and RIGHT (CGTCTGCGAGATCAACTACT) primers using the Go-Taq Flexi DNA Polymerase (Promega) followed by Sanger sequencing (Eurofins MWG, Ebersberg Germany).

**Figure 5:**
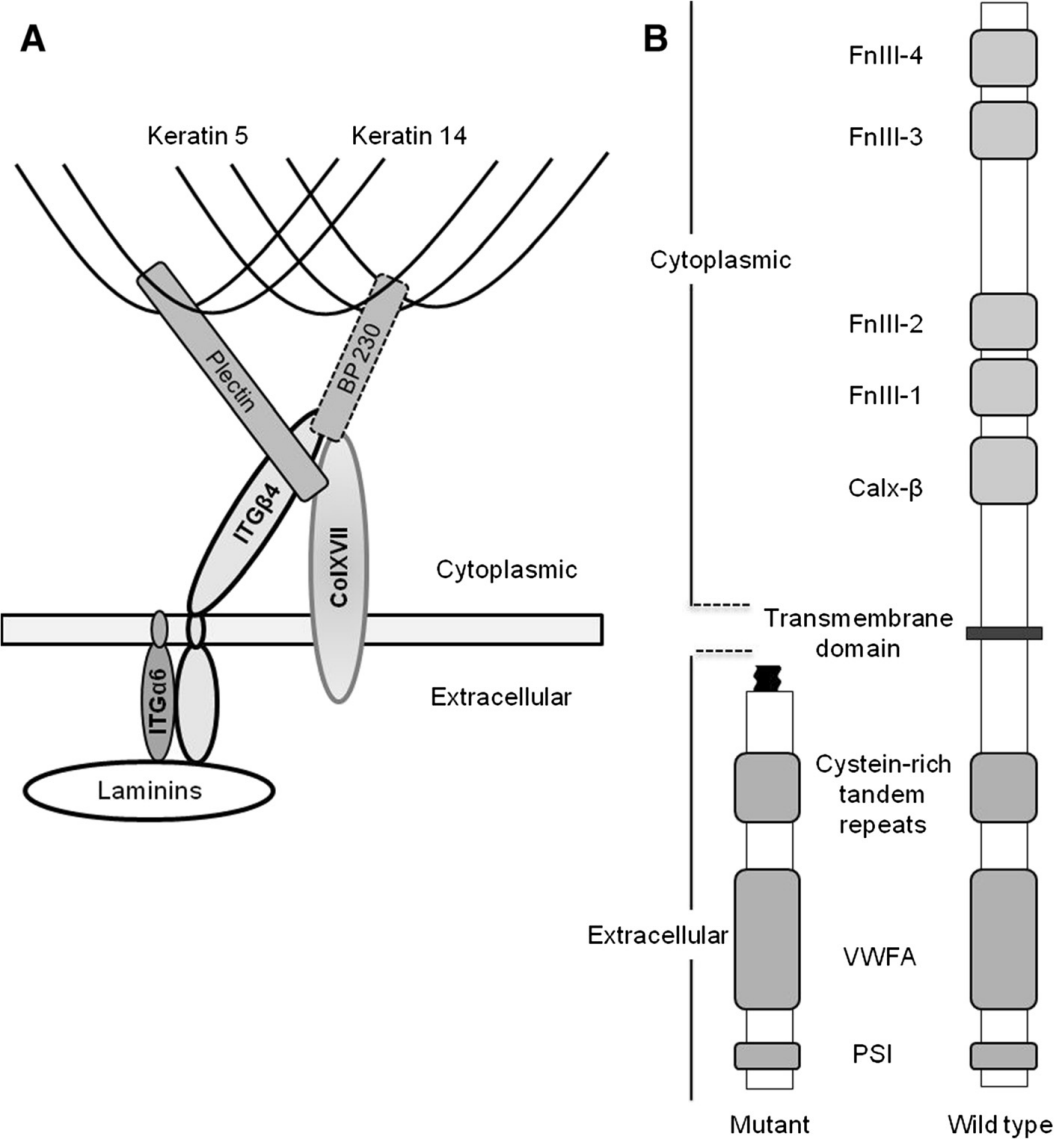
**Schematic representation of the components of the hemidesmosomes and of the** ***ITGB4*** **protein. (A)** Schematic representation of the components of the hemidesmosomes (adapted from http://xtal.cicancer.org/research.html). **(B)** Comparison between the structure of the mutant (predicted) and wild-type integrin β4 subunit (http://www.uniprot.org/uniprot/P16144). The wild-type integrin β4 comprises: (i) an extracellular region that contains the N-terminal plexin-semaphorin-integrin (PSI) and von Willebrand factor type A (VWFA) domains as well as a cysteine-rich repeat region; (ii) a transmembrane domain and (iii) a cytoplasmic region that includes a calx-beta (calx- β) domain and four fibronectin III-like domains (FnIII-1 to 4). The transmembrane domain and cytoplasmic region are predicted to be totally absent in the mutated β4 subunit.

*ITGB4* encodes the beta subunit of the alpha 6 beta 4 integrin heterodimer. This transmembrane receptor is a key component of the hemidesmosome anchoring complex which connects basal keratinocytes to the basement membrane by linking the extracellular N-termini of the α and β subunits to laminin 5 whereas the intracellular C-termini are attached to the cytokeratin network via plectin or via the type XVII collagen and the bullous pemphigoid antigen 1 (BP230) [17-21] (Figure 5A). In humans, numerous mutations in *ITGB4* have been reported to cause JEB with truncating mutations being associated with a more severe (and mostly lethal) phenotype [22]. In Charolais cattle, the deletion of exons 17 to 23 of *ITGB4* is predicted to result in a complete deletion of the transmembrane domain of the protein and the joining of exon 16 to exon 24 causes a frameshift leading to the production of a protein in which all the intracellular domains in addition to the transmembrane domain (*ITGB4* p.A665Gfs*11) are missing (Figure 5B). These extensive protein modifications are assumed to impair the association of *ITGB4* with *ITGA6* and other key proteins of the hemidesmosome anchoring complex. This is consistent with the absence of hybridization signals observed by Guaguere et al. [23] after immunohistochemical characterization of the skin of a JEB-affected Charolais calf with an antibody directed towards the alpha 6 beta 4 integrin complex.

**Figure 6:**
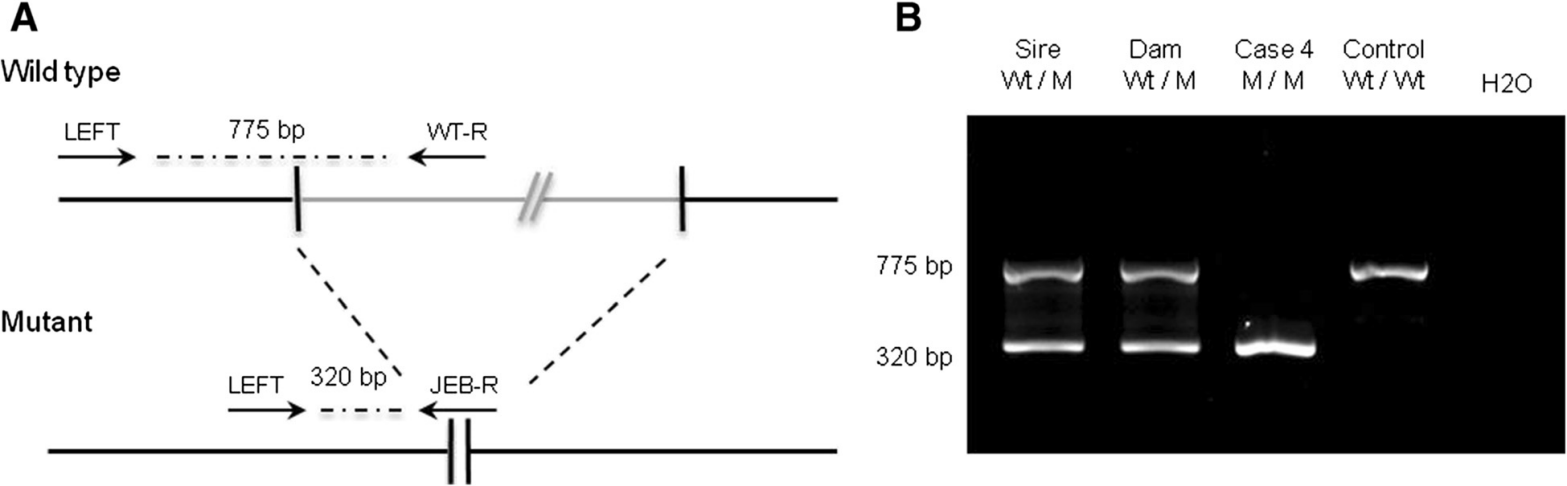
**Genotyping of the deletion using a 3-primer PCR system. (A)** Details on the design of the 3-primer PCR system. **(B)** Genotyping data of case 4, its parents, and a homozygous wild-type animal (based on haplotype information).

Genotyping of a second case available for DNA sampling (born at the end of our study) and of the six parents of the affected animals using a 3-primer PCR system (products amplified with primers LEFT, WT-R: TCTGCCCCACATGAATGCTT and JEB-R: AGACTGGGCACACAGACCAT using the same polymerase and revealed by electrophoresis on an ethidium-bromide-stained 2% agarose gel) revealed a perfect association between this mutation and the assumed genotypes of the individuals (Figure 6A and B).

Taken together, these arguments strongly support that this deletion within *ITGB4* is responsible for autosomal recessive JEB in Charolais cattle.

In an attempt to estimate the frequency of this mutation in the Charolais population that is bred by artificial insemination (AI), four parents were genotyped with the Illumina BovineSNP50 Beachip and phased as described in Boichard et al. [24] together with 6870 Charolais animals previously genotyped with the same array for genomic selection. Analysis of these data identified 44 animals that were all heterozygous for a rare (frequency = 3.2 %) 5.6-Mb segment (105 markers between positions 51 796 076 and 57 397 180 Mb on BTA19) that was shared identical by descent (IBD) with the haplotype carrying the *ITGB4* deletion. Surprisingly, subsequent genotyping of 16 of these animals with our 3-primer PCR system revealed that none were carriers of the deletion suggesting a rather recent origin for this mutation on the scale of the Charolais breed’s history. While our analysis also suggests the absence of the *ITGB4* deletion in the French AI population, it would be relevant to genotype the French natural mating population. Indeed the founder bull descends from animals exported to Great Britain at the end of the 1960’s from this population and it has been used as bull sire in French breeding herds in the 1990’s.

In conclusion, with only one case, we identified a mutation which appears to be necessary and sufficient to cause autosomal recessive junctional epidermolysis bullosa by exploiting the most recent DNA sequencing technologies. Targeted genotyping of at risk pedigrees with the genetic test that we developed will allow the rapid eradication of this rare genetic disease in Charolais cattle.

## Additional file

Additional file 1:
**Building of a local sequence assembly for the region deleted in the JEB calf.** This additional file provides details on the methodology used to build the local assembly of the deleted region in the JEB calf and the nucleotide sequence associated.
