# Supplementary material for: Whole-genome sequencing identifies a homozygous deletion encompassing exons 17 to 23 of the integrin beta 4 gene in a Charolais calf with junctional epidermolysis bullosa
Source: Genet Sel Evol. 2015 May 3;47(1):37. doi: 10.1186/s12711-015-0110-z (PMC4417276; doi:10.1186/s12711-015-0110-z)
Supplement: Additional file 1: — Building of a local sequence assembly for the region deleted in the JEB calf. This additional file provides details on the methodology used to build the local assembly of the deleted region in the JEB calf and the nucleotide sequence associated. [file 12711_2015_110_MOESM1_ESM.docx]

Since there are two gaps in the current bovine UMD 3.1 assembly within the region deleted in the JEB calf, we built a local assembly to determine the exact size and gene content of this deleted region.

First, a comparison was made between the two bovine assemblies by BLATing the deleted 4.8-kb region retrieved from the UMD3.1 (BTA19 g.56488278_56493087del) on the Btau 4 assembly using the UCSC genome browser (<http://genome.ucsc.edu>).

The gap located between positions 56488906 and 56489378 on UMD 3.1 was also present in the second bovine assembly. Thus, we performed chromosome walking from both sides of the gap taking advantage of the paired-end information and step-by-step manual alignments to fill this gap with a total of 166 base pairs using other WGS data available in our team [15].

The second gap (g.56492684-56492783 on UMD3.1) was absent from the Btau 4 assembly. Furthermore, comparative alignment of the two bovine assemblies revealed the presence of a duplicated segment that encompasses this gap in the UMD3.1 assembly (g.56492607-56493107 on UMD3.1). Subsequent alignment of the UMD3.1 assembly on the sheep, human and mouse genomes using the BLAT function in the UCSC genome browser (<http://genome.ucsc.edu>) suggested that this duplication was an artifact. Taking these observations into account, the region that covers the second gap and the duplication were removed and replaced by sequence of the Btau 4 assembly.

The final size of the mutation was determined by aligning the paired-end read from the JEB calf’s WGS on our assembly, leading to an exact deletion size of 2831 bp. For further confirmation, the newly built local assembly of the deleted region was BLATed on the sheep genome, which revealed an additional exon located between exons 20 and 21 at the level of the first gap and that consequently was not annotated in the UMD 3.1.

The newly built local assembly of the deleted region is presented below with the exact deleted region in JEB in capitals, exons in bold and the sequence part filling the gap underlined.

acagccctcctctcccatggGGCGGAGATGATGGAGATGGAAGTGGGGATGCGGGAGGTGGGGATAGGGGAGGTGGGACTTGGGGCTGGTACTGCCCACGGATGTGTCTTCGACTCAC**TTTTTCCCGGCGTTGGGCTGCTGC**CTGTGGGGGACAAAGTGAAGACTGTGAGCCAGATGGTCCCTAGGGGGCAAGGATTATCTGCTTGGGAGAAATCCCCTGGCATCAGCTATGGGAGCAGCGCCCCCCCCAACCAACCCCACCCCGGGCGCCTAGGACCCAC**CGGAACTTGGTCTGCTGGAGGTTGTGAGCGCCTGTGATTTGTCGGTACACCTCGTTCAGCTGCCAGGCAAGGGAGGGCTGTGAGGCTGGGGCCCTGGGGGAGCCCCTGCACCCAGAGGACCTCCTGGGCATGG**CTGGGGGTGTGGCCACAGGCATGTGCAGTGGCTCTACCCTTCTGGAAGCATTCATGTGGGGCAGATCCCAGGGGACCCCCCGGCCACACGTGTGTCCGAGAAAGCCCTTCTTTCCCACCCAAGGCGGCTTCCCTCTGCCCTCCTGCCACCCAGCTCRCACCCGGCCCAGAGGTGCCCACTGCCTGGGACCGGCTCCTTAC**ATTCTCCTCCACCTCCTGGCGCAGCTGGTCGCATTCTCGCGTGCCAGGCTTCAGCAGGTTCTCGGTGCAGAGGCGAGCAAGCCGCAGGGACAGTCCGTAGGGCACTAGGGCAGG**TGAGGAGGGGGGTCGTCAGTCCCGCCATGATGGGCACCACAGAAGCCTCCTCCAGAAGCCGGGCTCGGGATTGGGGGGCACCGGGGGCGTGTACCCCTCAGGAAGGGTAGGAGGTGGCCAGCCCTGTGCCTGCACCCGATACCCAGAGAAAAGAGGAATGCTGGATCCCAGACCGGGGAGCAGTGTGAATGTGCCCCGGGCCCCAAGGGCAGCGCCAGGAGACGTGGTGCCCAGCCCCGGCCCTCAC**CCAGCTCTGAGGGGTTGATGCCTGCAGCGTGCGAGGCGAAGCCCGGCCGCTGCACGTTGTTGGTGATCTTCCATCGGACTGTGTCGCGACCCTTGAGGTTCCCGCTGCGCAGCAAGGGCGTGTCCAAGTGGTCCGAGGCCATCAGGTTCTCTCGCAGCATGTAATGGTCTTCCTTGAAGCCCACCATGTGAC**CTGTGGGACCTGGGCCTCAGTACGGGGCTGAGGGGCTGCTTCCTAGACCCTCCCCCACCACACCCCACAGGTGGGACCTCCACCGTCCCCAGCCTCTGTCCCAGACTTGGCACCCTGCTTCCCCTCCACCCCCGCTGAAGGAAGGAGCTGTCAGAAGTCCACCGCCCCCGCCGAGGCCAGGCCCGTAC**CTCGGTTGCAGCAAGGGAGAAGGGCCAGGCAGGC**CTAGGAGGCAAGGAGGGCGGGGATGAGAGCTGAGACGGCCGCACCGGCCCAGGGAAGTCCCTCCGGCCACCGCCTCCCACCCACGCCAGTGGTCCAGAGGGGTCAGCAGTGCCTTCCCCAGACACTGGGATATCCAGGCAGTTACAGGGGCCTGGGTGTGGGCACAGAGGCGAACAGGGGCAGAGGGTACCCAAAATGCATGCCATGGGACTCTGGTCCCGCTATCTACTCTGGAAAGGAAAAGGTTCTGTGATCAAACCCCTTTGGGAAGCACTGAATTCCTTCCCCATGTAGTTTCCTTATGCAAACAGCTAGCTCAAAGGCTCCGAGAAGCCCTGCAGAGGAGAAACCGCTTTCTCAAAAGTGTTTGATCACAGAACGCTTTCTCCCTGTAACACGCATGAATACGACCCTGGGGGCTGCCGTAGGGGCACCTCTGCCCCCTTCAGTATCCCACGCCAGGCCATGCTCTGCACTCTCCTGTCTCCGGTCTTTCTTCCCAGGGAGATGGCCAGCTCCTGGTGGGCAGAGCCTTTCCTTCTTGTTGGTTCTCGTGACCCTTTGCATGCGGCTGACCCCCGGGCCAGGGACTCCCAGGAATCCAGCCACTGGTGGCTGGCAGAGGCGAGGGGCTGGGGTGGGGACGGCACCAGAGCTGGGCGGAACGTGTGGGTTCCCTTGGCCTCTGCCCCCTAGAGGGACCCCAGTGGGGTGGGGACCAGGGGAGCCCCCAC**CTTGCAGCAGGCACAGTACTTCCAGCAGAGCAGCAGCAGCAGGACCGGGAACAGGAGGAGGAAGATGAGCAGGGGGATGAGCCACCAGAAGGTGCCCGGGGGGCATT**CTAGGGAGGAGAGAGGAGGAGGAGGGAGCCAGTGCCTCCCCCCGATGTGGGTCTGACAGGCCACCCAGGCCCACCCTGCCAGCCCGCCGGCTCAC**CCTTCCTCCTCTGCACCAAGACGGTGCTGTTGGGCCCGGGGGCGCTGTCCCCCTCCACGGTGTAACTGTAGGTACAGTCGTCGTCCTCGTCCCGGAAGGAGCAGTGCTCCACCACCTCCTCCG**CTGCAGACACAGATGGTCAGCAGGGCACCCGACCCCAGCCCGGAGCACCTCAGGGCCTGGCGAGGGGTCCGCTCCTCACCACAGCCTTGCTCTGGGGCCCACCCTGCCCATCACAGGGTCAGGGTGGAGGGGTGGAGGCCTCTCCCTGGGGAAGAATGTGAGTTCCTGGCAGACTGTGCACACGGTGGGCGGTGAAGTGGGGGCTGCCCTGCCCAGGACCGTCAAAGCCCGCAAATCTGTGCCTGTCCTGGGCATCACTCAGGAGCTGGGCTTCTAGAACATTTTTCTGACCCAGAGGAAAGGAGGCTGTGTGCCCCAGGGCCCGTTTTTGCGCCTTTGCTATGTTCACGGCCCCCTCCCCAGGACTGGGTGCCCTGGCCAGACTGTGCGTCCCCTCCCTGGGtctgtgtgcccagtctgga
